# Elucidation of high permeability water among VACNFs using molecular dynamics

**DOI:** 10.1038/s41598-020-79596-1

**Published:** 2021-01-12

**Authors:** Ryosuke Matsuzaki, Yusuke Chisaka, Tomohiro Tajiri

**Affiliations:** Tokyo University of Science, 2641 Yamazaki, Noda, Chiba 278-8510 Japan

**Keywords:** Engineering, Mechanical engineering, Nanoscale materials, Carbon nanotubes and fullerenes

## Abstract

The cause of the high permeability in the flow of water in CNT (carbon nanotube)-based nanoscale materials remains to be elucidated. In this study, water impregnation simulations outside the VACNFs were performed using the molecular dynamics method to investigate the factors that cause high permeability by virtually changing the force field parameters. As a result, the permeability coefficient increased with increasing CNT content (*V*_*C*_) in the slip flow region. For the constant *V*_*C*_, the smaller the intermolecular force between water and CNTs, the higher the permeability coefficient. Because the intermolecular forces between water and CNTs are smaller than those between water and water, it may have an effect on the high permeability phenomenon. Furthermore, in the present *V*_*C*_ change, the arrangement structure of the water molecules changed from a disordered structure, such as bulk flow, to a chain structure in the impregnation direction, which is also considered a factor for the increase in the permeability. Therefore, both the intermolecular forces between water and CNTs and structural change in the arrangement of water molecules were factors in the high permeability phenomenon.

## Introduction

Vertically aligned CNT forests (VACNFs^1^) can be fabricated on a large scale at low cost using chemical vapor deposition. Their mesoporous structures have attracted attention owing to their potential applications in catalysis, nanofilters, and biosensors^2^. In recent years, CNT arrays such sas VACNFs have been applied to composite reinforcement materials^3^; however, the mechanism of resin impregnation in CNT arrays needs to be clarified. Moreover, the modeling and impregnation of the resin using molecular dynamics (MD) is difficult. Hence, water, which is the most basic fluid needed in the design of CNT-based nanofluidic devices, was treated in this study. Nanofluidic device design is concerned with the understanding and control of CNTs and fluid interactions. Therefore, the impregnation of water into the CNT was investigated in the analytical^4–7^ and Experimental^8–12^ sections. The flow and permeability coefficients were reported to be larger than those caused by the Hagen–Poiseuille relationship.

Among the experimental studies, Majumder et al.^9,10^ evaluated the penetration of water and various solvents into the MWNT (multi-walled CNT) membrane. Holt et al.^8^ evaluated the gas and water penetration and reported the flow and permeability coefficients that were several orders of magnitude greater than those of other solvents, gases, and commercial polycarbonates. Furthermore, their values showed an increase with decreasing fluid impregnation area, which was similar to that of the experimental results of Lee et al.^13^, who investigated the infiltration coefficient of water outside CNTs, specifically into VACNFs. To determine the cause of the specificity of water impregnation into CNTs, analytical studies using MD have been conducted.

Thomas et al.^5^ investigated the flow of water into the interior of CNTs and found that the permeability does not increase monotonically as the diameter of the CNT decreases, but that there is a region where it sharply decreases. The reason for this is the transition from the continuum to subcontinuum transport of the fluid. Walther et al.^7^ presented experimental evidence for these transitions with changes in the CNT diameter. Joseph et al.^14^ reported that the specificity of water in CNTs was due to the changes in water orientation and hydrogen bonding at the water–CNT interface.

As described above, analytical investigations using MD have been conducted to determine the cause of the high permeation flow and specificity of water impregnation into CNTs in comparison with other fluids; however, few studies have focused on the change in the molecular behavior between water and CNTs. In addition, although flow investigations in CNTs have been conducted analytically and experimentally, flow investigations outside CNTs have rarely been conducted. In the analytical investigation of the impregnation of external CNTs, water impregnation simulations in VACNFs were conducted to investigate the flow between CNTs ^15^. Moreover, the applicability of a continuous mechanics model based on the Gebart equation^16^ was derived using a assumed Hagen–Poiseuille flow with no-slip condition with respect to the permeability coefficient and evaluation of the flow trend in the nanosized pores have been investigated^15^. However, a physical explanation for the change in the trend and behavior of the molecules in the flow has not been provided. In addition, the dominant factor in the trend change has not been determined. Hence, it is necessary to understand the phenomenon from the perspective of both the water molecules and CNTs. Therefore, the purpose of this study is to verify the cause of the change in the flow tendency of each *V*_*C*_ (content of CNT) using molecular simulation with different conditions. In particular, as *V*_*C*_ increases, water molecules flowing between CNTs may be greatly affected by the CNT surface. Thus, the intermolecular forces between the CNT and water may contribute to the high permeability in high *V*_*C*_. However, the high permeability may also be due the change in the state of water molecules caused by the narrow space between CNTs. To clarify the effects of CNT and water molecules, the potential between CNT and water molecules was virtually changed.

## Results

### Fluid penetration outside the CNTs

The permeability coefficient formula according to the Darcy law [Eq. (1)], which is widely used to describe the permeation behavior of porous media, was used for evaluating the fluid penetration outside the CNT^17^.

1 $$ u_{\,MD} \,\,\, = \,\, - \,\,\frac{{K\,_{MD} }}{{(1\, - \,V_{C} )\,\mu \,_{MD} }}\,\,\frac{d\,P}{{d\,z}} $$

2 $$ \mu_{\,MD} \,\, = \,\,\frac{{k_{\,B} \,T}}{6\,\pi \,a}\,\frac{1}{D} $$

where *u*_*MD*_ is the fluid penetration velocity; *K*_*MD*_ is the permeability coefficient; *μ*_*MD*_ is the fluid viscosity; *dP*/*dz* is the pressure gradient in the flow direction; *a* is the molecular radius; *k*_*B*_ is Boltzmann's constant; and *T* is the absolute temperature. The viscosity was obtained using the self-diffusion coefficient *D*^18^ and Einstein’s equation^4^ expressed in Eq. (3), where *D* was obtained using the MD calculation.

3 $$ D\,\, = \,\,\mathop {\lim \,}\limits_{t\, \to \,\infty } \,\frac{1}{6\,t}\left\langle {\,\left| {\,{\mathbf{r}}\,(t\,^{\prime}\,\, + \,\,t)\,\, - \,\,{\mathbf{r}}\,(\,t\,^{\prime}\,)\,} \right|^{\,2} } \right\rangle \, $$

**Figure 1:**
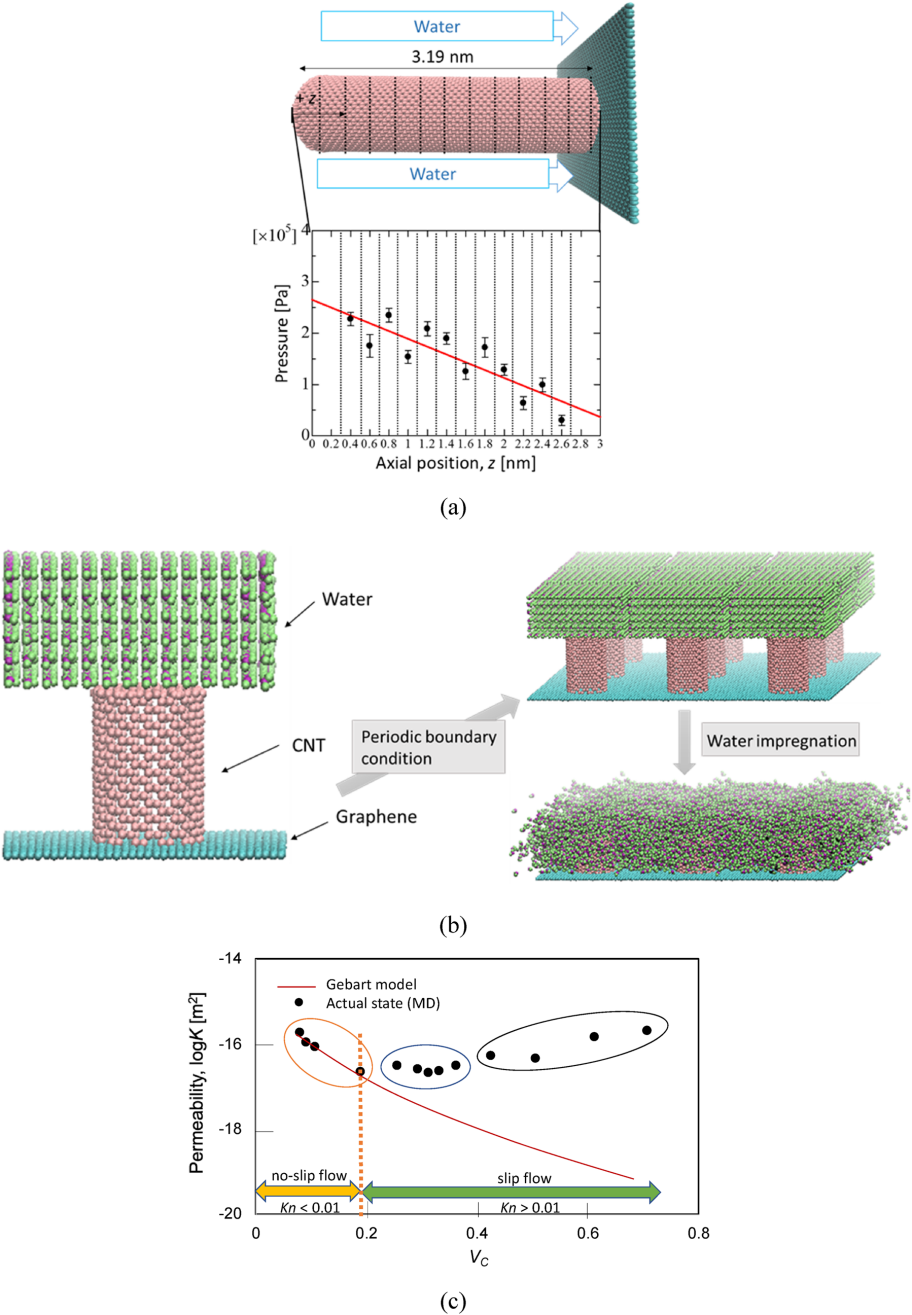
Permeability evaluation of water outside CNTs using MD^15^ (**a**) Axial pressure gradient between the CNTs at intervals of 0.2 nm. d*P*/d*z* was calculated by evaluating the pressure within several sub-volumes along the CNT axis and performing a linear regression analysis. (**b**) Analytical models and permeating behavior. VACNFs was made by applying the periodic boundary condition and permeating water outside CNTs. The figures (**a**) and (**b**) are created by VMD (version: 1.9.3, https://www.ks.uiuc.edu/Research/vmd/). (**c**) Permeability versus *V*_*C*_. The permeability was divided into three regions as a trend: region I (orange circle, *V*_*C*_ ≦ 0.106), the influence of the slip was small and the permeabilities decreased with increasing *V*_*C*_; region II (blue circle, 0.188 ≦ *V*_*C*_ ≦ 0.361), permeability increased or decreased with increasing *V*_*C*_; and region III (black circle, *V*_*C*_ ≧ 0.424), the area indicated the opposite tendency of region I.

**r**(*t*) is the position of the molecule at time *t*; *t*′ is the time origin; and the brackets $\langle \rangle$ are the autocorrelation. *dP*/*dz* was obtained using the following method: As shown in Fig. 1a, the VACNF was divided into regions with 0.2 nm intervals in the range of 0.1 to 2.7 nm, and the pressure was calculated to be the value in the center of the region (0.2, 0.4, …, 2.6 nm). Then, a graph of the position and pressure in the CNT axis was drawn, and the slope of the graph was given as *dP*/*dz* using linear approximation. In MD, the pressure was calculated using the virial theorem^19^ expressed in Eq. (4).

4 $$ P\,\, = \,\,\frac{{N\,\,k_{\,B} \,\,T}}{V}\,\, + \,\,\frac{1}{3\,\,V}\,\,\left\langle {\,\,\sum\limits_{i\, > \,j}^{N} {\,\,\sum\limits_{{}}^{N} {\,\,{\mathbf{r}}_{\,ij} \,\, \cdot \,\,{\mathbf{F}}_{\,ij} } } } \right\rangle \,\,,\,\,\,{\mathbf{r}}_{\,ij} \,\, = \,\,{\mathbf{r}}_{\,i} \, - \,\,{\mathbf{r}}_{\,j} $$

where *V* denotes the volume in the region; *N* is the number of atoms; **r**_*ij*_ is the distance between atoms *i* and *j*; and **F**_*ij*_ denotes the interaction force acting between atoms *i* and *j*.

### Analytical model

As shown in Fig. 1b, the analytical model consists of a CNT with a cap in the center of graphene and water molecules. The diameter and length of the CNTs were 2.16 nm and 3.19 nm, respectively, and the size of the graphene was varied in order that *V*_*C*_ = 0.077, 0.090, 0.106, 0.188, 0.254, 0.291, 0.311, 0.33, 0.361, 0.424, 0.505, 0.611, and 0.706. The total number of atoms in the system varied according to the *V*_*C*_ and ranged from 6099 to 42,939. Here, *V*_*C*_ was obtained as *V*_*C*_ = (area of CNT)/(area of graphene) in the model in Fig. 1b.

**Figure 2:**
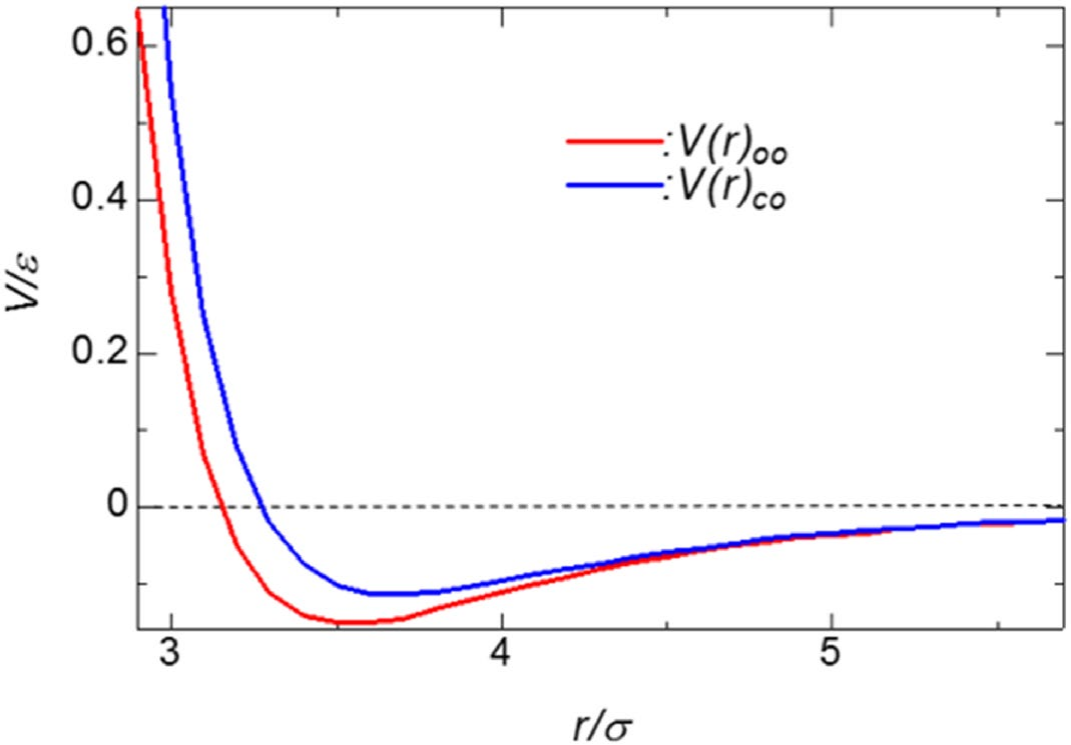
Theoretical curves of terms representing van der Waals interactions in the intermolecular force field of the potential function AMBER96.

Figure 2 shows the theoretical curve of the term representing the van der Waals interaction in the intermolecular force field of the potential function AMBER96, where the theoretical equation is given by Eq. (5).

5 $$ V\left( r \right) = 4\varepsilon \left\{ {\left( {\frac{\sigma }{r}} \right)^{12} - \left( {\frac{\sigma }{r}} \right)^{6} } \right\} $$

where ε is the depth of the potential well; σ is the distance at which the potential is zero; and *r* is the distance between the two particles. In addition, Thomas et al.^5^ investigated the flow of water in a CNT and reported that one of the reasons for the increase in the average flow rate and permeability with decreasing CNT diameter was because water molecules were no longer attached to the CNT surface, resulting in a decrease in flow friction. Therefore, in order to verify this, the change in the permeability coefficient was observed by changing ε_CO_, which is the intermolecular force between water and CNTs.

### MDS and calculation results

Figure 1c shows the relationship between the permeability coefficients with respect to *V*_*C*_, which were the Knudsen number *Kn* < 0.01 when *V*_*C*_ < 0.188 and *Kn* > 0.01 when *V*_*C*_ ≥ 0.188. In this figure, the actual permeability coefficient using the MD result for *V*_*C*_ ≥ 0.188 increased with increasing *V*_*C*_, but the permeability coefficient (*K*_*G*_) for the no-slip state, calculated from the continuum mechanics model, decreased. This trend was consistent with the experimental results of Lee et al.^13^, who investigated the permeability of water into the VACNF. On the other hand, as the impregnation area became wider, that is, in the range of *Kn* < 0.01, the two values were consistent. From this trend, it can be said that there is an evident discrepancy between the trends in the range of *Kn* > 0.01. In addition, as *V*_*C*_ changes, there can be three regions: (I) a region where the effect of slip is small and the permeability decreases as *V*_*C*_ increases (orange circles), (III) a region showing an opposite trend (gray circles), and (II) a region where the permeability increases or decreases as *V*_*C*_ increases (blue circles).

Therefore, the trend of the permeability coefficient obtained from the analysis was discussed. It was assumed that the increase or decrease in the permeability coefficient was affected by the diffusion in the CNT axial direction, that is, in the impregnation direction. Moreover, it was discussed that changes in the arrangement structure of water during the impregnation process occur, causing fluctuations in the diffusion. In addition, it was predicted that the intermolecular forces between water and CNTs may have an effect. Therefore, we focused on three points: diffusion along the CNT axis, visualization of the arrangement structure of water, and change in the intermolecular forces between water and CNTs.

**Figure 3:**
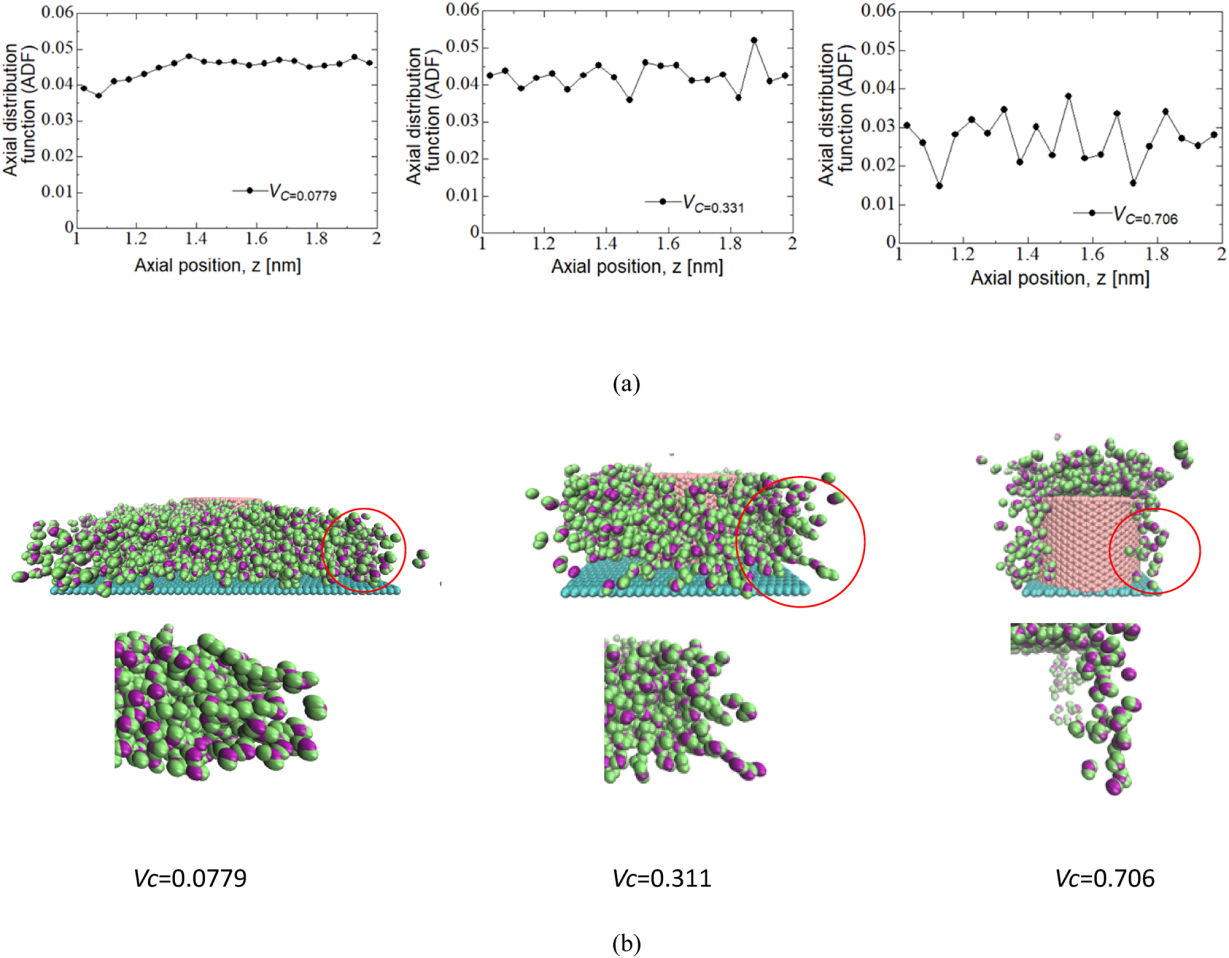
(**a**) Relationship between the axial position (between 1.0 nm and 2.0 nm) and ADF (axial distribution function), and water structure outside the CNT in the 0.0779, 0.311, 0.706-*V*_*C*_ VACNFs. (**b**) Water molecule arrangement just after water permeates to VACNFs completely. The figure is created by VMD (version: 1.9.3, https://www.ks.uiuc.edu/Research/vmd/).

First, the axial diffusion of the CNTs was described. The axial distribution function (ADF)^5^ was introduced and calculated for the water molecules among the CNTs in the VACNF to measure the trend change of the flow. The ADF (*a*(*z*)) is defined as the number of water molecules between *z* = *L*_1_ and *z* = *L*_2_, *N*_*t*_ in the CNT axial direction and is expressed as the following equation:

6 $$ \begin{gathered} \int_{{\,L\,_{1} }}^{{\,L\,_{2} }} {a(z)} \,Adz = N_{t} - 1 \approx N_{t} \hfill \\ \Rightarrow \int_{{\,L\,_{1} }}^{{\,L\,_{2} }} {a(z)} dz = \frac{{N_{t} }}{A} \hfill \\ \end{gathered} $$

where *A* is the area of the water-impregnated area. In this study, we divided the CNTs into regions with an interval of 0.05 nm in the range of 1.0. 2.0 nm in length, and determined the number of water molecules in the region after water impregnation using. MD. We set the value at the center of the region (1.025, 1.075, …, 1.975 nm). Figure 3a shows the ADF results for *V*_*C*_= 0.0779, 0.311, and 0.706. Based on the trend of the graph, the transition of the graph was relatively smooth at *V*_*C*_ = 0.0779 with no sharp peaks observed. Whereas the transition was not smooth at *V*_*C*_ = 0.706 with different peaks observed. Moreover, for *V*_*C*_ = 0.331, the two trends were mixed. From these results, it can be seen that as *V*_*C*_ increases, the flow changes from a gentle trend to a trend with many peaks. Therefore, when *V*_*C*_ is small, the ADF is approximately constant regardless of *z*, and water molecules exist in a disordered manner without a specific arrangement. When *V*_*C*_ is large, the ADF varies depending on *z*, and the ease of diffusion of molecules locally changes.

Next, as shown in Fig. 3b, the arrangement structure of the water molecules was examined, and at *V*_*C*_ = 0.0779, the water had a disordered arrangement structure like a bulk flow. On the other hand, at *V*_*C*_ = 0.706, it had a straight chain shape. For *V*_*C*_ = 0.331, the trend was between 0.0779 and 0.706. Therefore, it can be observed that the sequence structure of the *V*_*C*_ changes to a chain structure in which the molecules were arranged in the direction of the CNT axis as *V*_*C*_ increases.

**Figure 4:**
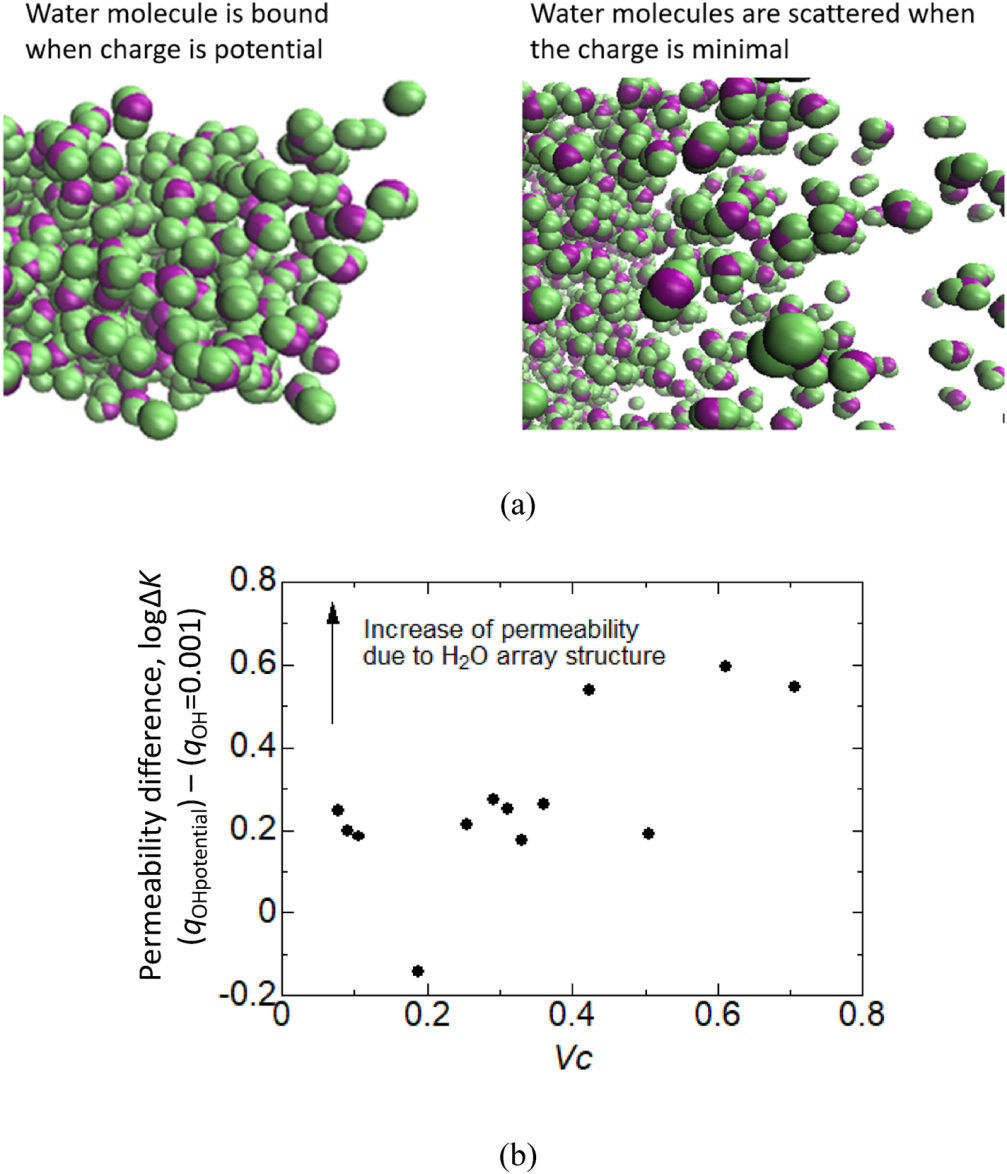
Effect of the arrangement structure of water molecules. (**a**) Image of water molecule structure with potential and 0.001 charges. The figure is created by VMD (version: 1.9.3, https://www.ks.uiuc.edu/Research/vmd/). (**b**) Permeability difference when the electric charge was 0.001 and the potential charge of water molecule.

Next, to evaluate the effect of the arrangement structure, it was reduced by setting the charge among the water molecules to a small value (*q*_OH_ = 0.001) as shown in Fig. 4a. As a result, a change in the permeability coefficient was observed. Figure 4b shows the difference between the charge of the potential and penetration coefficient for each *V*_*C*_ for *q*_OH_ = 0.001. The results showed that the difference in permeability for the actual and small charges was not zero, but rather a difference with a correlation coefficient of 0.68. The difference increased with increasing *V*_*C*_.

**Figure 5:**
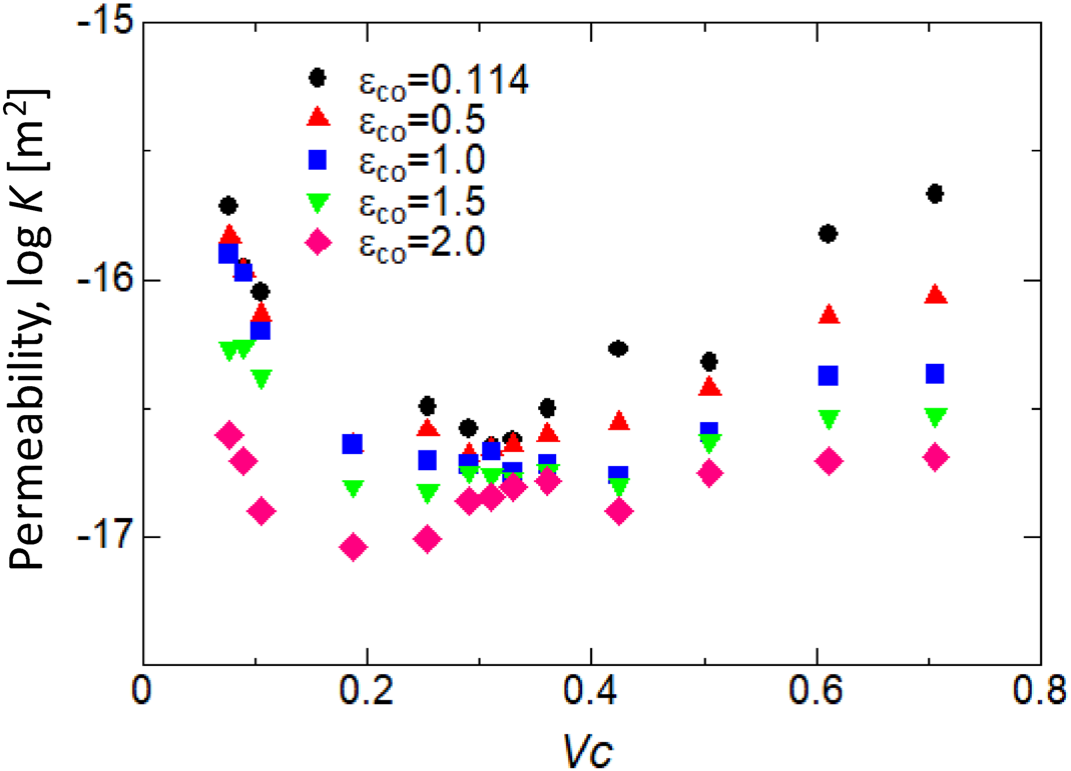
Results of the MD simulation: *V*_*C*_ versus permeability (log*K*) in *ε*_CO_ = 0.114, 0.5, 1.0, 2.0; we changed the *ε*_CO_ to clarify its influence on permeability when changing intermolecular force.

As for the intermolecular force between water and CNTs, it changed with changing ε_CO_, and the permeability coefficient changed at a constant *V*_*C*_. In this study, five ε_CO_s were investigated: 0.114, 0.5, 1.0, 1.5, and 2.0. Figure 5 shows the results of the permeability coefficients for each ε_CO_. From this figure, it can be seen that the trend of the permeability coefficients for *V*_*C*_ was the same when ε_CO_ was changed, and the overall permeability coefficients decreased with increasing ε_CO_.

**Figure 6:**
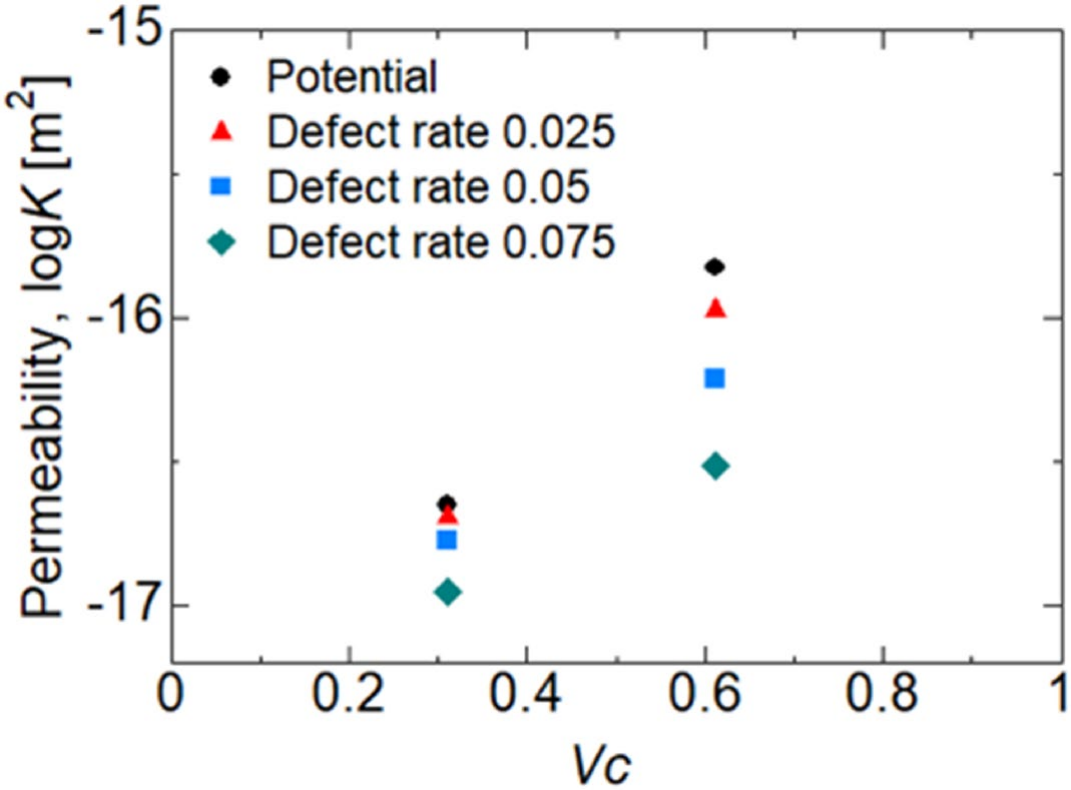
Water permeability in the interspace between CNTs with various carbon atom defect ratios.

The actual CNTs contain defects (i.e., lack of carbon atoms) or functional groups. The effects of the ratio of the number of defects in the CNTs on permeability were analyzed, and shown in Fig. 6. Although increased permeability with increasing *V*_*C*_ was observed for all defective CNTs, the permeability decreased with increasing defect rate, suggesting that irregularities on the surface of the CNTs may be a potential source of flow resistance. Other factors, such as doping of CNTs, water purity, irregular distance between CNTs, etc., will be investigated in the future.

## Discussion

**Figure 7:**
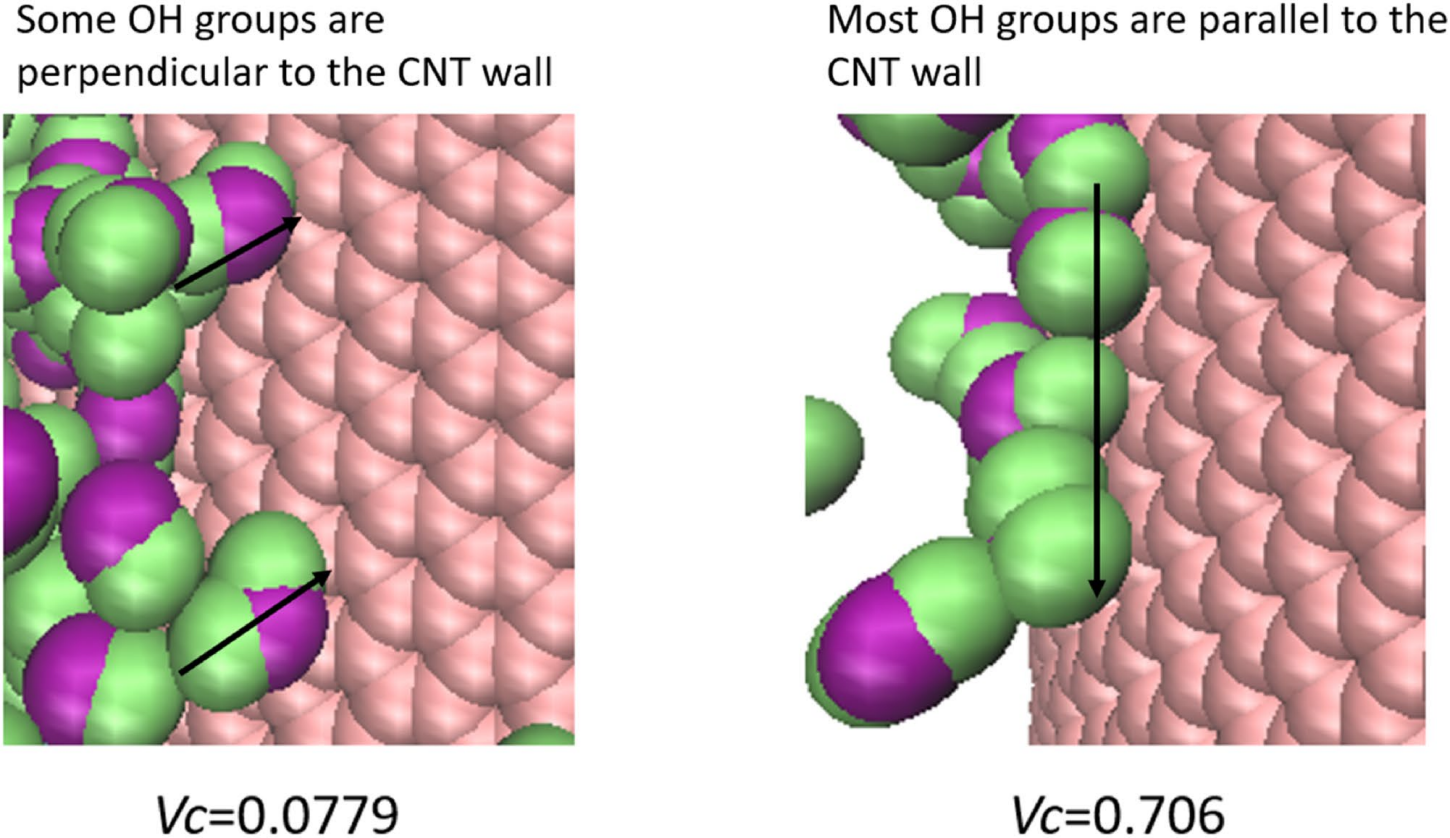
Comparison of OH groups facing the CNT wall.

The results of the ADF and arrangement structures of the water molecules showed that the structure of the water molecules changes from the bulk to linear states as *V*_*C*_ increases. In addition, Joseph et al.^14^ suggested that the orientation of water with free OH bonds towards the CNT surface and the decrease in the number of hydrogen bonds at the water–CNT interface may cause the high permeability phenomenon. Considering this, when the impregnation area was large, the OH group of the water molecule faced the CNT surface and became stable, which led to an increase in the number of hydrogen bonds and decrease in the permeability. On the other hand, the narrower the impregnation area, the more restricted the orientation of the water molecules were. The more fixed they were, the fewer OH groups facing the wall and fewer hydrogen bonds. This led to an increase in the permeability. Figure 7 shows the magnified images of water molecules near the wall of *V*_*C*_ = 0.0779 and *V*_*C*_ = 0.706. It can be seen from this figure that the number of OH groups facing the wall decreased with increasing *V*_*C*_.

Next, the positive correlation coefficient for the difference in the permeability coefficients in Fig. 4 indicated that the penetration promotion effect of the water arrangement was stronger when the flow channel was narrow. This suggested that the high penetration phenomenon due to the arrangement of water molecules was also related to the interaction with the CNT surface.

A comparison of the change in the intermolecular force between water and CNTs in Fig. 5 showed that the smaller intermolecular force between water and CNTs led to an increase in the permeability coefficient. As shown in Fig. 2, the intermolecular force in the potential state is smaller between water and CNTs than that between water and water. This result physically confirmed Thomas et al.^5^′s discussion that one of the reasons for the increase in the mean flow velocity and permeability coefficient with decreasing CNT diameter was that water molecules are no longer attached to the CNT surface, which results in a decrease in flow friction.

**Figure 8:**
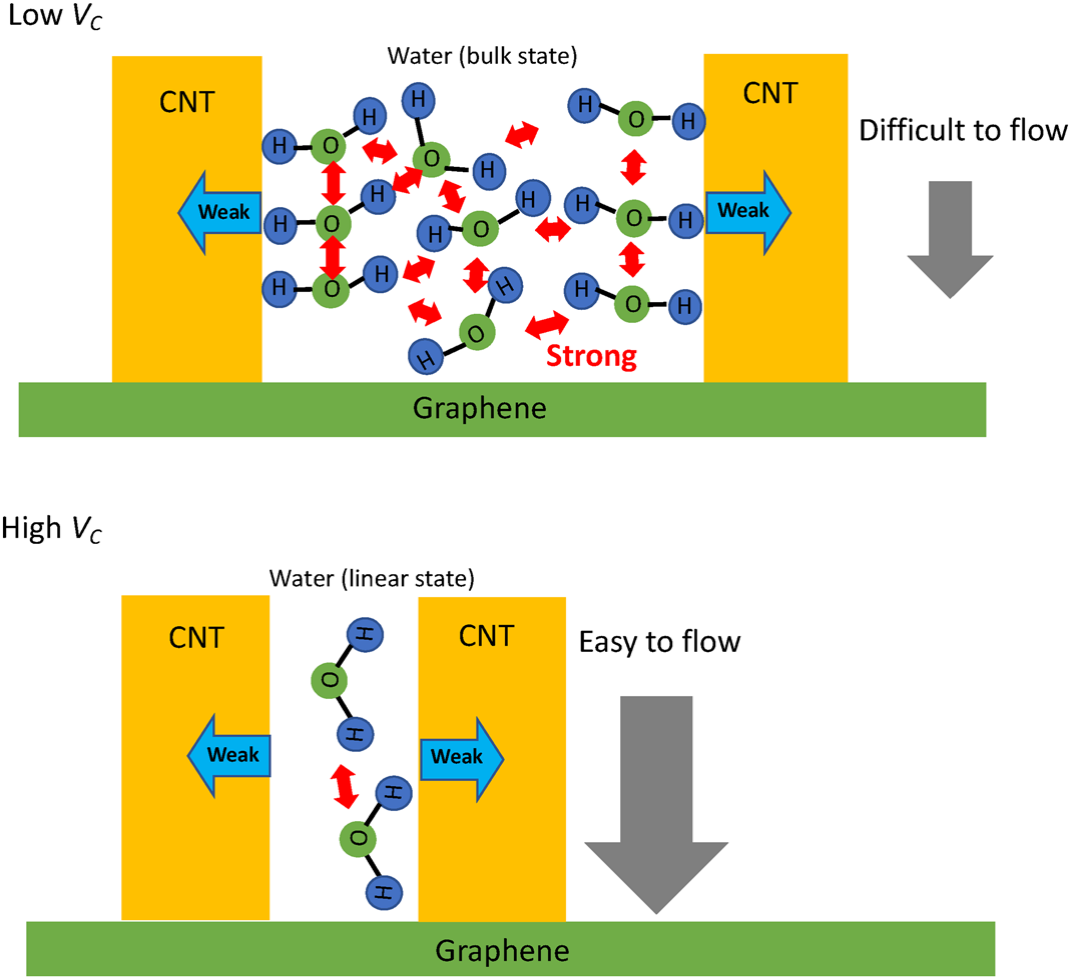
Mechanism of increase in water permeability in the interspace of CNTs in high *V*_C_.

These results suggested that two factors contribute to the high permeability phenomenon: the change in the arrangement structure from the bulk to linear structures during nanoscale material penetration, and the small intermolecular forces between water and CNTs (Fig. 8).

## Methods

### Molecular dynamics simulation

In this study, MDS was performed using a general-purpose molecular dynamics software, Large-scale Atomic Molecular Massively Parallel Simulator (LAMMPS), to impregnate the external CNTs in VACNF with water. The TIP3P model^20–22^, AMBER96^23^, and MD was used for the water model, potential function, and the viscosity of water, respectively. The particle–particle-particle mesh (PPPM)^24^ and SHAKE methods^25^ were used to calculate the long-range Coulomb interaction, and constrained equations of motion with a mean square error of 0.0001, respectively. The relaxation calculations in the MDS were performed by solving the Langevin equation of motion^26^ to control the temperature, and introducing the NVE ensemble to control the volume and energy. The NVT ensemble with Langevin's equation of motion was also used in the impregnation simulation. Here, the flow should be steady when the diffusion coefficients are obtained by the Green–Kubo linear response relation^27,28^. Because the impregnation simulation deals with unsaturated flow, the diffusion coefficient should not be obtained during this time. Therefore, in this study, we considered the flow after the water molecules were fully impregnated to be stationary, and calculated the diffusion coefficient at that time. The parameters in the potential function of AMBER96 are listed in Table 1 and the calculation conditions for the relaxation, impregnation, and diffusion coefficient simulations are shown in Table 2.

**Table 1:** Parameters of the AMBER96 potential function.

Atoms(*ij*)	*ε*_*ij*_ [kcal/mol]	*σ*_*ij*_ [Å]
CC	0.086	3.407
OO	0.1521	3.151
HH	0.0	0.0
CO	0.114	3.275
CH	0.0	0.0
Bonds(*ij*)	*K*_*ij*_^*R*^ [kcal/mol/Å^2^]	*R*_*ij*_^*eq*^ [Å^2^]
OH	1000.0	1.0
Angles(*ijk*)	*K*_*ijk*_^*θ*^[kcal/mol/rad^2^]	*θ*_*ijk*_^*eq*^ [°]
HOH	1000.0	109.47

**Table 2:** Computational condition of the simulation.

Relaxation	Timestep [fs]	0.01
	Running time [fs]	20
	Damping constant	100.0
Simulation (permeation)	Timestep [fs]	1.0
	Running time[fs]	8.0 × 10^5^
	Temperature[K]	300
Simulation (calculating self-diffusion coefficient)	Timestep [fs]	1.0
	Running time [fs]	4.0 × 10^5^
	Temperature [K]	300
